# Activation of Anterior Insula during Self-Reflection

**DOI:** 10.1371/journal.pone.0004618

**Published:** 2009-02-26

**Authors:** Gemma Modinos, Johan Ormel, André Aleman

**Affiliations:** 1 BCN Neuroimaging Center, University of Groningen, Groningen, The Netherlands; 2 Interdisciplinary Center for Psychiatric Epidemiology (ICPE), Department of Psychiatry, University Medical Center Groningen, University of Groningen, Groningen, The Netherlands; University of Granada, Spain

## Abstract

**Background:**

Functional neuroimaging studies have suggested activation of midline frontoparietal brain regions to be at the core of self-related processes. However, although some studies reported involvement of the insula, little attention has been paid to this region as forming part of the “self”-network.

**Methodology/Principal Findings:**

Using functional magnetic resonance imaging (fMRI), we aimed at replicating and extending previous studies by scanning subjects whilst reflecting upon their own personal qualities as compared to those of an acquaintance. A third condition with statements about general knowledge was used to control for attention, semantic processing and decision making processes. The results showed a significant effect of task in brain activity, consistent with previous findings, by which both person conditions recruited a common set of medial prefrontal and posterior regions, yet significant differences between self and other were found in the medial prefrontal cortex (MPFC) and the anterior cingulate cortex (ACC). Notably, significant neural activation in the left anterior insula was observed as uniquely associated with self-reflection.

**Conclusions/Significance:**

The results provide further evidence for the specific recruitment of anterior MPFC and ACC regions for self-related processing, and highlight a role for the insula in self-reflection. As the insula is closely connected with ascending internal body signals, this may indicate that the accumulation of changes in affective states that might be implied in self-processing may contribute to our sense of self.

## Introduction

The study of the neural substrates of self-related processing has become an increasingly prominent issue in cognitive neuroscience. Recent functional neuroimaging studies have consistently shown brain activity within several cortical regions during self-referential tasks, particularly along the midline and including the prefrontal cortex (PFC), posterior cingulate cortex (PCC), and parietal regions. Moreover, these regions appear to be involved across a variety of task domains, namely self-reflection [1], [2], encoding and retrieval of self-related information [3]–[6], self-related processing in sexual arousal [7], assessment of one's own personality and physical traits [8]–[11], self-referential decision-making [12], and first-person perspective taking [13]. Critically, these relevant regions for social cognition strikingly overlap with the neural substrates of the so-called “default mode” of the human brain [14]. In this regard, the accumulating evidence for the existence of a default mode of brain function has derived from the observation of intrinsic activity in a neural architecture encompassing regions of the MPFC, PCC and medial precuneus [15], whose interaction would be intimately involved in self-awareness and conscious experience [16]–[18].

Efforts to closely examine neural activity associated with self-referential as opposed to other-person processing have described activity in MPFC, anterior cingulate cortex (ACC), supplementary motor area (SMA) and precuneus for self versus a famous other [2], [9], [11], ventral MPFC for self versus a similar or dissimilar other [19], and superior medial parietal and MPFC for self versus a friend [4], [20]. Recently, functional magnetic resonance (fMRI) evidence was examined in a meta-analysis by Northoff et al. [21], which concluded in favor of a neural module of cortico-subcortical midline structures underlying self-processing. Conversely, a thorough review of the available behavioral and physiological evidence of the possible specificity of a sense of self postulated that the observation of brain activations associated with self-processing might not be indicative of neural specificity due to methodological irregularities such as the use of inadequate control conditions (e.g., lack of another person condition; the use of baseline conditions based on case judgments, requiring a dissimilar cognitive process). Instead of indicating self-relatedness, the authors proposed that such activations would rather be attributable to generally processing information about people [22].

The insular cortex is well known for its role in interoceptive awareness and appraisal of one's own internal states [23]. Furthermore, this structure has been involved in studies employing affective tasks, being traditionally associated with pain [24]. The insular cortex expands bilaterally along the innermost parts of the human forebrain, being neuroanatomically plausible to distinguish between the anterior and posterior portions. The anterior insula, corresponding to the agranular cortex, is thought to be involved in social cognition [25]. Several fMRI studies on self-related processes reported insula activity [10], [12], [17], [26]–[30], yet little elaboration was made of such finding. Hence, the present study placed special emphasis on whether the anterior insula would have a role in a more general and in principle non-emotion-eliciting self-reflection task.

Understanding the neural regions supporting self-reflection, a crucial component of self-awareness, could be critical to elucidate the intrinsic brain mechanisms whose impairment may lead to psychological disturbances [31]. Impaired self-awareness is a common feature in several neurologic [32]–[34] and psychiatric disorders, such as schizophrenia [35], [36], in which prefrontal and cingulate regions are frequently compromised [37]. Interestingly, recent findings suggest that impairments in the default mode network may also be associated with psychiatric disorders [38].

The aim of this study was two-fold: 1) to clarify whether insula activity would be intrinsically involved in self-reflection, and 2) to replicate and expand current understanding of the neural network underlying self-reflection using an fMRI task previously associated with robust activity in midline structures [1]. Considering that Johnson and colleagues' study [1] used a semantic baseline, and was therefore prone to criticism [22], we sought to address this issue by adding an other-person reflection condition.

Based on previous data, we hypothesized that: 1) both person conditions (compared to the semantic baseline) would show some common activation patterns in prefrontal and parietal regions [5], [11], [20]; 2) self-reflection (compared to other-reflection) would still produce unique activations within the cortical midline, especially in its anterior portion, and including the anterior insular cortex.

## Materials and Methods

### Participants

Sixteen right-handed, healthy university students (10 male, aged 18–27 years; *M* = 20.8 years, *SD* = 2.5) participated in the study. Subjects did not have a history of psychiatric or neurological disorder, significant physical illness, head injury, or alcohol/drug abuse. None of the subjects were taking alcohol or medication at the time of the study. All subjects scored below cut-off on self-reports of depression [39] (*M* = 2.13, *SD* = 2.05), and psychosis-proneness [40], (*M* = 1.12, *SD* = 0.04). These tests were used to rule out interfering factors associated with psychopathology that may affect self-awareness [41]. After subjects were given a complete explanation of the study, written informed consent was obtained from all of them. Participants were paid for their participation. The study was approved by the Medical Ethical Committee of the University Medical Center Groningen, and was conducted in accordance with the Declaration of Helsinki.

### Task

Subjects viewed three types of short sentences about personal qualities, attributes and attitudes. They were asked to indicate whether the sentence was true about them, whether it was true about an acquaintance of theirs, or whether it was true about general knowledge. Before scanning, a detailed explanation of the task was given to all subjects and they were asked to choose an acquaintance upon whom they were to reflect inside the scanner. An eligible acquaintance was defined as someone familiar to the degree of being able to judge some of their personality and physical traits, but not too close and not eliciting strong feelings in them. Thus, best friends, parents or romantic partners were not permitted; instead, an acquaintance such as a classmate or a teammate was suggested.

### Procedure

The experiment comprised 90 trials, 30 per condition, including items such as, for the Self condition, “I am a good friend”, “I often forget important things”, “I am attractive”, “I am good at my studies”. Sentences in the Other condition involved, for example, “OTHER can be trusted”, “OTHER is a generous person”, “OTHER is not very attractive”, “OTHER often talks too much”. The Semantic condition (baseline) included sentences such as “You need water to live”, “A vertebra is a bone”, “A hand has 8 fingers”, “Spring comes after Autumn”. There were an equal number of true/false items in the semantic condition (50% of each), and of physical/personality traits in both person conditions (27% physical, 73% personality-related) so that results would not be confounded by this effect. Similarly, there were the same number of positive and negative key-words in the two person-related sets of sentences (50% of each), validated against Anderson's list of personality trait words [42], in order to control for a possible effect of valence on brain activity. Subjects responded to each statement, presented in white letters on a black screen, with a “yes” (right hand, index finger) or “no” (right hand, middle finger) button-press response. A constant visual reminder of which button to press was displayed at the bottom of the screen throughout the entire scanning session.

The experimental design consisted of 5 blocks for each of the 3 conditions (Self, Other, Semantic), interleaved with four 20-s rest periods consisting of a fixation cross presented in the middle of the screen (at the beginning, middle and end of experiment). Order of presentation of the 3 conditions was randomized. In each 24-s block, 6 different sentences of the same type were presented for 4 s each.

The experiment was executed on a Pentium PC using the software package E-Prime (Psychology Software Tools Inc., Pittsburgh, PA). Stimuli were projected onto a screen positioned at the end of the bore, visible through a mirror attached to the head-coil. Cushions were used to minimize head movement.

### Imaging data acquisition

Images were acquired with a 3T Philips Intera MR-scanner (Philips Medical Systems, Best, The Netherlands). Functional data comprised 246 volumes acquired with T2*-weighted gradient echo planar imaging (EPI) sequences, using a sense-8 head coil. Thirty-seven echo planar images per volume sensitive to blood oxygenation level-dependent (BOLD) contrast were obtained (TR = 2000 ms; TE = 35 ms; 3.5×3.5 mm in-plane resolution; Field of View [FOV], 224 mm). Slices were acquired interleaved and oriented parallel to the AC-PC plane, with a thickness of 3.5 mm and no gap. High-resolution T1-weighted 3D fast-field echo (FFE) sequences were obtained for anatomical reference (160 slices; TR, 25 ms; TE, 4.6 ms; slice-thickness, 1 mm; matrix, 256×256; FOV 26 cm; voxel size, 1×1×1 mm).

### Imaging data analyses

The fMRI data were preprocessed and analyzed using SMP5 (Wellcome Department of Imaging Neuroscience, London, UK) implemented in Matlab 7.1 (The Mathwork Inc.). All functional images were slice-time corrected, and realigned. After realignment, the obtained mean EPI image was co-registered with the structural T1 image. Subsequently, images were spatially normalized to the standard stereotactic space defined by the Montreal Neurological Institute (MNI) template. During normalization, scans were re-sampled onto a 2×2×2 mm^3^ grid. Functional images were spatially smoothed with a 3D isotropic Gaussian kernel (FWHM of 8 mm). Low-frequency noise was removed by applying a high-pass filter (cut-off of 128 s) to the fMRI time-series at each voxel. Significant hemodynamic changes for each condition were examined using the general linear model [43], with a blocked (epoch) design modeled using a boxcar function convoluted with a hemodynamic response function. Statistical parametric maps for each contrast of the *t*-statistic were calculated on a voxel-by-voxel basis for each subject (first-level analysis on Self>Semantic and Other>Semantic).

The contrast images obtained per subject were entered as the data for the second-level (group) analyses. In order to examine significant brain activation in each effect, we used a one-way within-subjects analysis of variance (ANOVA). One experimental factor was specified, with two levels (Self>Semantic, Other>Semantic). Peak coordinates were identified at *p*<0.05, False Discovery Rate corrected (FDR), in regions encompassing at least 10 voxels. After determining the main effect of task, we examined differences between self and other, by means of a *t*-test between the two levels (e.g., [self>semantic]>[other>semantic]), setting the same statistical correction for multiple comparisons as mentioned above.

## Results

### Behavioral data

Reaction times (RT) during the Self (M = 1917 ms), Other (M = 1986 ms), and Semantic conditions (M = 1937 ms) did not differ significantly from each other (F(2, 45) = 0.318; p = 0.729). This was considered as indicative that none of the three conditions might have required a superior cognitive effort, which enabled a clearer interpretation of results.

No significant differences were found when examining the proportion of the participants' attribution of positive qualities to self or to other (F(1,30) = 0.119; p = 0.773). All subjects scored above 90% correct in the semantic condition (M = 28.19, SD = 1.11, maximum: 30).

### Functional imaging results

**Figure 1:**
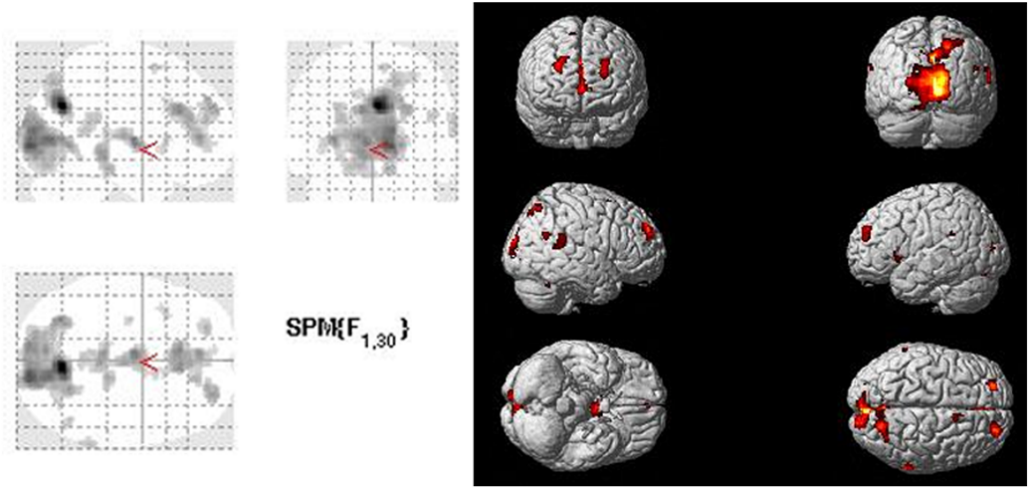
Glass brain and rendered view depicting the main effect of task, as characterized with a one-way within-subjects ANOVA. Significant differences were recognized at *p*<0.05 (FDR corrected) and extent threshold of 10 voxels.

The within-subjects analysis revealed that there was a main effect of task in the precuneus extending into the superior parietal lobe, in the ACC, and bilaterally in the middle frontal and superior frontal gyri (MPFC). Additional activations were seen in the superior temporal gyrus (STG), calcarine gyrus, SMA, and the left anterior insula (Table 1 and Figure 1).

**Table 1:** Neural activation produced by the main effects of task.

Region	L/R	MNI Coordinates			*F-value*	*Z*-score
		*x*	*y*	*z*		
Anterior Cingulate Cortex	L	−8	28	28	29.56	4.35
	R	6	28	28	28.31	4.28
Middle Frontal Gyrus	R	26	54	30	25.09	4.08
	L	−24	52	32	17.85	3.53
Superior Frontal Gyrus	R	20	52	36	22.80	3.92
	L	−24	50	22	21.14	3.80
Precuneus	R	4	−62	32	62.53	5.65
Calcarine Gyrus	R	14	−88	0	42.48	4.97
Superior Temporal Gyrus	L	66	−40	18	18.10	3.55
SMA	R	8	10	62	17.03	3.46
Insula	L	−36	20	4	15.87	3.35

SMA = Supplementary Motor AreaActivations were identified with a *t*-test between [self>semantic]>[other>semantic], and vice-versa. Significance was set at *p*<0.05 (FDR corrected), encompassing at least 10 voxels.

**Figure 2:**
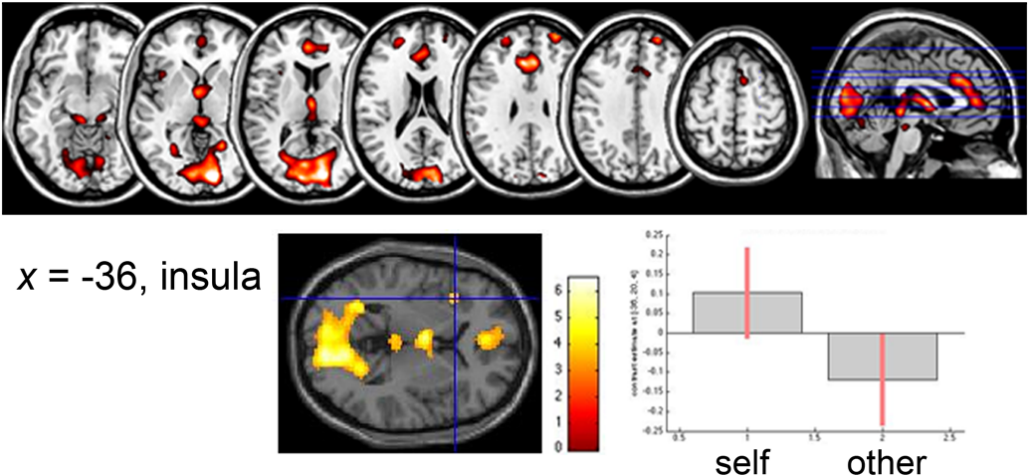
Brain regions with significantly greater activation for Self-reflection, identified at *p*<0.05, FDR corrected, extent threshold of at least 10 voxels. Activation in the insula is depicted in the sagittal view, plotting its differential activation as obtained with the *t*-test conducted on the within-subjects design ([*Self>Semantic*]>[*Other>Semantic*]). Bars depict variance loadings and 90% confidence intervals.

The *t*-test analysis between the two levels ([self>semantic]>[other>semantic]) indicated that the Self condition produced significant neural activation in ACC, middle and superior frontal gyri (MPFC), STG, SMA, and the left anterior insula. In addition, brain activity was seen in the calcarine gyrus, and the cerebellum. (Table 2 and Figure 2).

**Table 2:** Brain activation differentially associated with Self and Other conditions.

Region	L/R	MNI coordinates			*T*-score	*Z*-score
		*x*	*y*	*z*		
***Self-reflection***						
Anterior Cingulate Cortex	L	−8	28	28	5.44	4.50
	R	6	28	28	5.32	4.43
Middle Frontal Gyrus	R	26	54	30	5.01	4.24
	L	−24	52	32	4.22	3.71
Superior Frontal Gyrus	R	20	52	36	4.77	4.09
	L	−24	50	22	4.60	3.97
Superior Temporal Gyrus	R	66	−40	18	4.25	3.73
SMA	R	8	10	62	4.13	3.64
Insula	L	−36	20	4	3.98	3.54
***Other-reflection***						
Precuneus	R	4	−62	32	7.91	5.77
Superior Parietal Lobe	R	20	−68	50	4.68	4.02

SMA = Supplementary Motor Area. Activations were identified with a *t*-test between [self>semantic]>[other>semantic], and vice-versa. Significance was set at *p*<0.05 (FDR corrected), encompassing at least 10 voxels.

**Figure 3:**
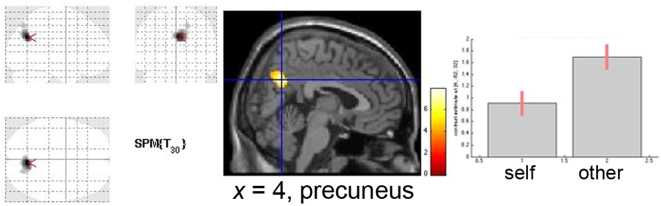
Brain regions with significantly greater activation for Other-reflection, identified at *p*<0.05, FDR corrected, extent threshold of at least 10 voxels. Activation in the precuneus is depicted in the sagittal view, plotting its differential activation as obtained with the *t*-test conducted on the within-subjects design ([*Other>Semantic*]>[*Self>Semantic*]). Bars depict variance loadings and 90% confidence intervals.

Regarding the Other-reflection condition, the reverse *t*-test analysis ([other>semantic]>[self>semantic]) showed a significant effect in the right precuneus, and the right superior parietal lobe. (Table 2 and Figure 3).

In sum, Self-reflection significantly explained brain activity in a neural architecture comprising the left anterior insula, MPFC and ACC, along with temporal and occipital regions.

**Figure 4:**
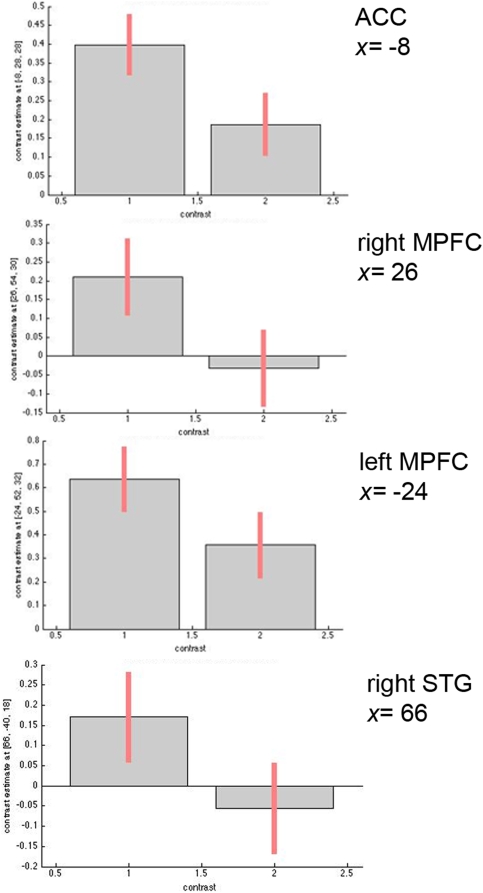
Significant differences in activation within the anterior cingulate cortex (ACC), medial prefrontal cortex (MPFC, left and right), and superior temporal gyrus (STG) obtained with the *t*-contrast on the within-subjects analysis of variance ([*Self>Semantic*]>[*Other>Semantic*]). 1 indicates Self>Semantic; 2, Other>Semantic. Activations were considered significant at *p*<0.05 FDR corrected. Bars depict variance loadings and 90% confidence intervals.

Finally, we examined the effects of interest in these regions in order to ascertain the direction of these associations. These contrasts revealed that, at *p*<0.05 (FDR corrected), the significant difference in insula activation was produced by Self rather than Other (F(3,30) = 4.625; p = 0.040). As for the MPFC, ACC, and STG, we observed that activation in those regions significantly increased when subjects engaged in self-reflection rather than when they reflected upon an acquaintance or upon general knowledge (right MPFC, F(1,30) = 6.484, p = 0.016; left MPFC, F(1,30) = 4.904, p = 0.035; ACC, F(1,30) = 7.591, p = 0.010; STG, F(1,30) = 5.388, p = 0.027) (Figure 4). Finally, variability in the precuneus was greater during the Other person condition (F(1,30) = 9.827; p = 0.004).

## Discussion

This study investigated whether the extent to which self-reflection modulates brain activity could be dissociable from reflecting upon an acquaintance and upon general semantic knowledge, with special focus on the involvement of the insular cortex. The analysis revealed self-related activity in a neural architecture comprising the anterior MPFC and ACC. Notably, the anterior insula showed unique activity associated with self-reflection.

Particular interest was placed on the role of the insula in self-reflection. The insular cortex is predominantly regarded as associated with interoceptive awareness [23], emotional salience [44], awareness over subjective feeling [45] and bodily arousal states [46], and has been reported in self-referential tasks in the emotional domain [21]. Given that the present experiment was not designed to elicit explicit emotional feeling states (compared to, e.g., studies employing emotional pictures), our results could suggest a more general function of this area in self-referential processing. Alternatively, considering its well-known role in emotion [47], insular activity could actually be related to an emotional component that might be inherent to self-processing; in other words, to the self being an emotional entity *per se*. Indeed, definitions of emotion emphasize that an emotion is caused by a person consciously or unconsciously evaluating an event as relevant to their concerns or goals [48], [49]. Thus, evaluating one's own personal characteristics, as in the present self-reflection task, would almost by definition involve emotion. In addition, as the insula is closely connected with ascending internal body signals [50], the accumulation of changes in affective states that might be implied in self-processing may contribute to our sense of self. Taken together, this evidence supports the notion that the insula is a supramodal structure, involved in a myriad of processes closely related to facilitating a sense of self. Disturbances in insula activity may result in social and emotional impairments commonly observed in psychopathology, as recently suggested by a study showing differential insula involvement in the failure to maintain adequate social interactions by people with Borderline Personality Disorder [51].

The MPFC activity associated with self-processing aligns well with prior research [1], [4], [5], [7]–[9], [11], [12], [19], [29], [52], [53], [54]. This study was designed to provide an improvement for the limitations that had been claimed from those previous studies [22]. The fact that reaction times in the paradigm used herein were longer than in studies using single trait adjectives [2], [4], [8], [9], [12], [29] could indicate that comparing self and other in terms of personal attitudes, traits and attributes helped induce a more elaborate reflective process on one's own sense of self and identity. Hence, these data expand the findings from Johnson et al. [1] by showing distinct recruitment of the MPFC for self-reflection not only when compared to general knowledge but also when controlling for the neural activity associated with reflecting upon another person. Furthermore, the MPFC is crucial in what has been proposed as a default mode of self-evaluation [55], in which this region would support automatic evaluation of self [17]. Beyond the evident overlap between our findings and previous research in default mode activity, the concurrent insula activation observed herein expands prior knowledge on which regions within the human forebrain are key players in self-awareness. Gusnard et al. [17] reported left insula activity when subjects reflected upon their emotional state, therefore supporting this area's role in emotional self-awareness. We showed that anterior insula activity is associated with reflecting upon non-explicitly emotional stimuli, suggesting its involvement in a distributed network of neural regions, the activation of which is increased when specifically attending to self-referential mental activity. Similarly, self-reflection also induced differential ACC activity, which aligns well with previous work [4], [7], [42], [54], [56]–[58], and is suggestive of this area's greater engagement for self- than for other-person reflection.

The present report failed to replicate the findings from Johnson et al. [1] of a specific involvement of the PCC in self-reflection. However, their study did not use an other-person reflection condition. In our study, the precuneus, a neighboring parietal region to the PCC, did show a strong BOLD response as a main effect of task, which aligns well with its role in representing person characteristics [1], [8]–[10], [59]. These results may thus support the involvement of posterior areas in perspective-taking [13] and comparison processes [60], as subjects might have employed comparison and simulation when reflecting upon an acquaintance. Moreover, further testing confirmed that its activation was largely explained by other-reflection. The precuneus involvement in self-processing tasks has been related to its role in autobiographical memory [16]. Thus, as a tentative explanation, the greater precuneus activity for Other found in the present study might have been induced by the subjects' need to draw on autobiographical memories about shared occasions in the past in order to judge the acquaintance's personal and physical attributes.

Finally, the STG was also recruited for self-reflection. The STG has been described as important for social cognition, particularly relevant in the Theory of Mind network [for review, see 61]. The differential activation found in our experiment supports the notion that brain regions involved in self-relatedness are also intrinsically participative in social cognitive processes, in line with simulation accounts [53], [56].

This study could not control for social desirability, therefore subjects could have been biased towards endorsing false responses as veridical in order to give a better image of themselves. However, it was emphasized to subjects before scanning that the experiment was about thinking about their true characteristics, and that the experimenters had no interest whatsoever in their personal qualities. Unfortunately, the risk that subjects may respond with a positive bias might be inherent to paradigms on self-relatedness and social cognition. Nonetheless, we considered the fact that RTs were not significantly longer for any of the three conditions as indicative that no differential psychological process was interfering with one specific condition (e.g., thinking about how I want to be instead of how I am; increased uncertainty when judging person attributes compared to general knowledge). Furthermore, subjects did not attribute more positive items to themselves than to the other person, which further confirmed that there was no apparent interference of stimuli valence in brain response. In light of the difficulty to rule out the possibility of untruthful responses, future similar paradigms could benefit from the inclusion of a measure of social desirability. Finally, the present study was not designed to examine the distinction between physical and mental self on brain activity. It is therefore possible that the results reported herein reflect brain activity involved in processing personality characteristics to a greater extent, since they were used more often. However, both conditions comprised an equal number of both types of traits, so that this would not affect the contrast between Self and Other-reflection. Future studies specifically designed to this end may help expand current knowledge on the cerebral areas involved in reflecting upon different types of person characteristics.

In sum, the results from the present study shed new light on the functional roles of the anterior insular cortex by showing its involvement in self-reflection. Our results confirmed previous evidence of a relative overlap within the neural systems subserving Self- and Other-related processing (as shown by the main effect of task), yet some independent regions for self-reflection were identified – MPFC, ACC and anterior insula. Thus, rather than merely reflecting general person-processing, as suggested before [22], there is accumulating evidence for a set of anterior cortical regions intimately involved in self-related processing. Future research in schizophrenia and in disorders of emotion regulation such as alexithymia [62] with a focus on these areas could help determine the nature of the lack of awareness that is frequently present in these disorders.
